# A novel mitochondrial amidoxime reducing component 2 is a favorable indicator of cancer and suppresses the progression of hepatocellular carcinoma by regulating the expression of p27

**DOI:** 10.1038/s41388-020-01417-6

**Published:** 2020-08-18

**Authors:** Dehai Wu, Yan Wang, Guangchao Yang, Shugeng Zhang, Yao Liu, Shuo Zhou, Hongrui Guo, Shuhang Liang, Yifeng Cui, Bo Zhang, Kun Ma, Congyi Zhang, Yufeng Liu, Linmao Sun, Jiabei Wang, Lianxin Liu

**Affiliations:** 1 Department of Hepatic Surgery, Key Laboratory of Hepatosplenic Surgery, Ministry of Education, The First Affiliated Hospital of Harbin Medical University, Harbin, 150001 Heilongjiang China; 2 Department of Colorectal Surgery, Harbin Medical University Cancer Hospital, Harbin, 150001 Heilongjiang China; 3 Division of Life Sciences and Medicine, Department of Hepatobiliary Surgery, Anhui Province Key Laboratory of Hepatopancreatobiliary Surgery, The First Affiliated Hospital of USTC, University of Science and Technology of China, Heifei, 230001 Anhui China

**Keywords:** Liver cancer, Liver cancer, Prognostic markers, Prognostic markers

## Abstract

Hepatocellular carcinoma (HCC) is the fifth leading cause of cancer-related mortality in the United States. Exploring the mechanism of HCC and identifying ideal targets is critical. In the present study, we demonstrated metabolism dysfunction might be a key diver for the development of HCC. The mitochondrial amidoxime reducing component 2 (MARC2) as a newly discovered molybdenum enzyme was downregulated in human HCC tissues and HCC cells. Downregulated MARC2 was significantly associated with clinicopathological characteristics of HCC, such as tumor size, AFP levels, and tumor grade and was an independent risk factor of poor prognosis. Both in vitro and in vivo studies suggested that MARC2 suppressed the progression of HCC by regulating the protein expression level of p27. The Hippo signaling pathway and RNF123 were required for this process. Moreover, MARC2 regulated expression of HNF4A via the Hippo signaling pathway. HNF4A was recruited to the promoter of MARC2 forming a feedback loop. MARC2 levels were downregulated by methylation. We demonstrated the prognostic value of MARC2 in HCC and determined the mechanism by which MARC2 suppressed the progression of HCC in this study. These findings may lead to new therapeutic targets for HCC.

## Introduction

Hepatocellular carcinoma (HCC) is the fifth leading cause of cancer-related mortality in the United States [1]. The major risk factors for HCC are hepatitis B, hepatitis C, alcohol, and metabolic syndromes [2]. HCC has been classified into subsets with distinct molecular and clinical features by genomic studies [3]. TP53 mutation as one of the most common oncogenic drivers of HCC defines the HCC subtype in terms of high frequency of vascularization, invasion, and poor differentiation [4]. However, these subsets have not been addressed therapeutically. Therefore, the mechanism underlying HCC requires further investigation.

The mitochondrial amidoxime reducing component 2 (MARC2) is a newly discovered molybdenum enzyme, which forms a N-hydroxylated reduction system together with CYB5R and CYB5 catalyzing the reduction of N-hydroxylated compounds, such as amidoximes [5, 6]. This reduction system could also generate nitric oxide (NO) from nitrite and may be involved in the pathway of NADH-dependent hypoxic NO production [7, 8]. Elevated MARC2 was involved in the process of differentiation of murine 3T3-L1 cells, whereas its downregulation impaired lipid synthesis [9]. Recently, certain studies have shown that MARC2 was downregulated in cancer tissues, such as breast and colon, and was associated with the onset of colon cancer [10, 11]. However, the role of MARC2 in cancer is still unclear.

In the present study, we aimed to explore the role of MARC2 in the progression of HCC. MARC2 was downregulated in HCC tissues and was significantly associated with clinical characteristics and patient survival. MARC2 suppressed the progression of HCC by preventing the degradation of p27. The Hippo signaling pathway and RNF123 were required for this process. MARC2 was downregulated by methylation. These data indicated that MARC2 might be a tumor suppressor with regard to the progression of HCC.

## Results

### Metabolic dysfunction may play a key role in the development of HCC

**Fig. 1:**
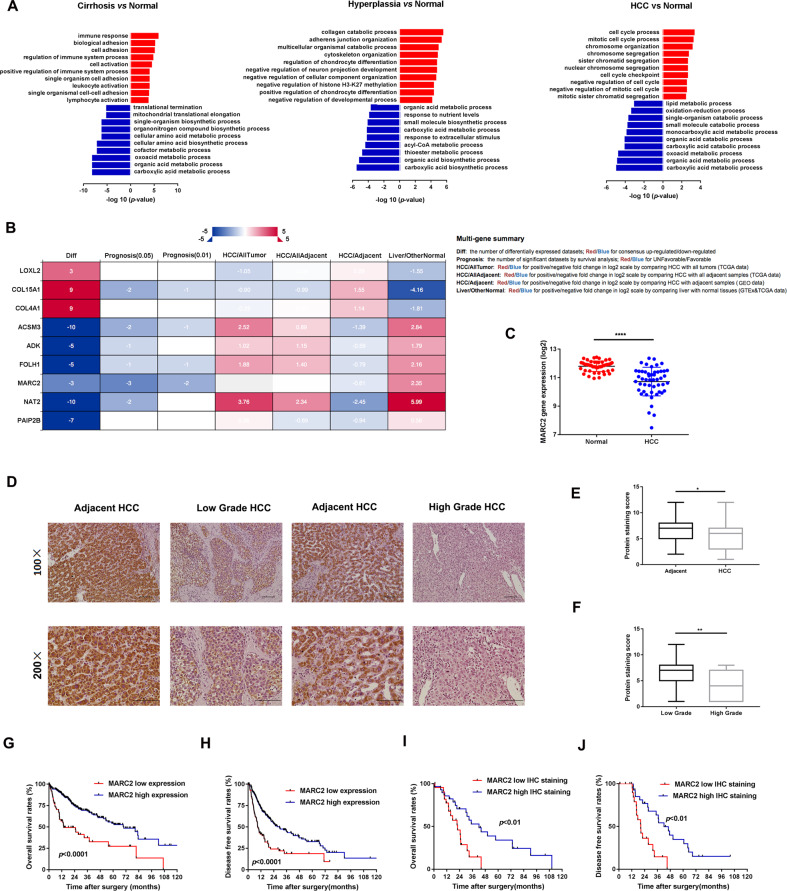
MARC2 is downregulated in HCC and predicted poor prognosis. **a** Gene expression data were extracted from GSE3500, GSE6764, and GSE14520. Different expression gene existed in all the three databases were filtered and separated into two groups (upregulated group: *red* and downregulated group: *blue*) enriching in cell process. **b** Filtered genes were valid in different databases in the left panel; the annotation for column in the datasheet in the right panel. The survival analysis of MARC2 was significant in three datasets (prognosis *p* < 0.05), and more significant in two datasets (prognosis *p* < 0.01). **c** MARC2 mRNA levels were significantly decreased in HCC tissues (*n* = 50) compared with the paired adjacent normal tissues (*n* = 50). **d** Representative images of MARC2 IHC staining in human HCC tissues and adjacent normal liver tissues. Bar = 100 µm (*100); Bar = 50 µm (*200). **e** IHC staining score was used to compare the protein expression of MARC2 between HCC tissues (*n* = 57) and the adjacent normal tissues (*n* = 82). **f** IHC staining score was used to compare the protein expression of MARC2 between patient with low tumor grade (*n* = 36) and patient with high tumor grade (*n* = 19). **g**, **h** The Kaplan–Meier method was used to compare the OS and DFS between the patient group with high level of MARC2 and patient group with low level of MARC2. **i**, **j** The Kaplan–Meier plots of OS and DFS between patients with high (*n* = 15) and low level of MARC2 (*n* = 15) according to IHC scores. The log-rank test was used to compare survival between different groups. *p < 0.05; **p < 0.01; ***p < 0.001, all of which were considered statistically significant.

In order to investigate the mechanism for the formation of HCC, we combined the following three GEO databases: GSE3500, GSE6764, and GSE14520, which contained normal, cirrhotic, hyperplasia, and HCC samples, representing the key stages in the development of HCC. The gene expression data of the cirrhosis, hyperplasia, and HCC groups were compared to those of the normal group. The genes with differential expression were separated into two groups (upregulated and downregulated) and enriched into the cell process. The cell processes in the upregulated group were different during the process of HCC development, while the changes noted in the downregulated group were consistent (Fig. 1a). The major cell processes in the downregulated group were involved in the cell metabolism, such as carboxylic acid metabolic and organic acid metabolic (Fig. 1a). These data indicated that dysfunction of normal metabolism may serve as a key driver for the development of HCC. Subsequently, the differentially expressed genes, which participated in all the processes from the transition of normal liver to HCC and were present in all the three databases, were filtered and validated in the TCGA and GEO databases. Finally nine genes were selected including MARC2 that exhibited a stronger association with patient prognosis (Fig. 1b).

### MARC2 is downregulated in HCC and can predict poor prognosis

MARC2 exhibited particularly high expression in liver tissues (Fig. S1A) and was downregulated in different types of tumors including HCC (Fig. S1B). TCGA mRNA microarray analysis of HCC patients indicated that the gene expression of MARC2 was significantly decreased in tumor tissues compared with that of the adjacent non-tumor tissues (Fig. 1c). MARC2 downregulated in HCC tissues was also detected by different GEO and ICGC-LIRI databases (Fig. S1C). The analysis of the clinicopathological characteristics indicated that lower expression of MARC2 was significantly associated with larger tumor size, higher levels of AFP, and higher tumor grade (Table 1). Cox regression analysis indicated that low expression level of MARC2 was an independent risk factor for both OS and DFS of HCC patients (Tables 2, 3). Subsequently, we examined the protein expression levels of MARC2 in a tissue microarray cohort of 87 patients with HCC by IHC staining (Fig. 1d). The expression level of MARC2 was lower in HCC tissues than that noted in adjacent non-tumor tissues, whereas MARC2 staining exhibited lower intensity in HCC patients with higher tumor grade (Fig. 1e, f). Kaplan–Meier analysis indicated that the gene expression levels of MARC2 correlated positively with patient survival. Patients with higher MARC2 expression levels indicated better overall survival (OS) and disease free survival (DFS) (Fig. 1g, h). The same prognostic analysis results had also been confirmed by the GSE14520 and ICGC-LIRI-JP databases (Fig. S1D). Patients with higher protein expression of MARC2 were also inclined to present with better prognosis compared to those with lower expression of MARC2 (Fig. 1i, j). These data indicated that MARC2 might serve as a favorable indicator for the progress of HCC.

**Table 1:** Clinic characteristics.

	MARC2 gene expression		
	Low expression	High expression	*p* value
Age(years)			
≤60	68	64	0.636
>60	76	80	
Gender			
Male	95	100	0.529
Female	49	44	
AJCC			
I	72	77	0.574
II + III + IV	62	56	
Tumor size			
≤3	78	104	**0.011**
>3	106	81	
Lymph node			
Yes	55	53	0.882
No	129	132	
Metastasis			
Yes	46	58	0.215
No	138	127	
Tumor grade			
G1 + G2	60	94	**<0.001**
G3 + G4	74	39	
AFP			
≤400	88	115	**<0.001**
>400	46	18	

The bold values are significant *p*-value.

**Table 2:** Overall survival.

	Overall survival			
	Univariate		Multivariate	
	HR (95% CI)	*p* value	HR (95% CI)	*p* value
Age	1.014 (0.999–1.029)	0.052	1.010 (0.995–1.025)	0.199
Gender	1.192 (0.830–1.714)	0.342		
AJCC	1.644 (1.343–2.012)	**<0.001**	1.090 (0.539–2.218)	0.804
Tumor size	1.657 (1.382–1.985)	**<0.001**	1.440 (0.718–2.873)	0.306
Lymph node	1.507 (1.039–2.187)	**0.031**	0.909 (0.553–1.495)	0.708
Metastasis	1.721 (1.186–2.495)	**0.004**	1.880 (1.160–3.038)	**0.01**
Tumor grade	1.130 (0.889–1.435)	0.318		
AFP	1.060 (0.648–1.741)	0.810		
MARC2	0.737 (0.649–0.836)	**<0.001**	0.770 (0.672–0.882)	**<0.001**

The bold values are significant *p*-value.

**Table 3:** Disease free survival.

	Disease free survival			
	Univariate		Multivariate	
	HR (95% CI)	*p* value	HR (95% CI)	*p* value
Age	0.998 (0.985–1.010)	0.731		
Gender	1.120 (0.809–1.550)	0.496		
AJCC	1.722 (1.442–2.057)	**<0.001**	1.958 (0.974–3.938)	0.059
Tumor size	1.660 (1.413–1.949)	**<0.001**	0.796 (0.396–1.597)	0.520
Lymph node	1.265 (0.906–1.765)	0.168		
Metastasis	1.243 (0.892–1.733)	0.199		
Tumor grade	1.143 (0.933–1.399)	0.197		
AFP	1.095 (0.725–1.655)	0.666		
MARC2	0.779 (0.684–0.887)	**<0.001**	0.781 (0.645–0.946)	**0.011**

The bold values are significant *p*-value.

### MARC2 suppresses the progression of HCC both in vitro and in vivo

**Fig. 2:**
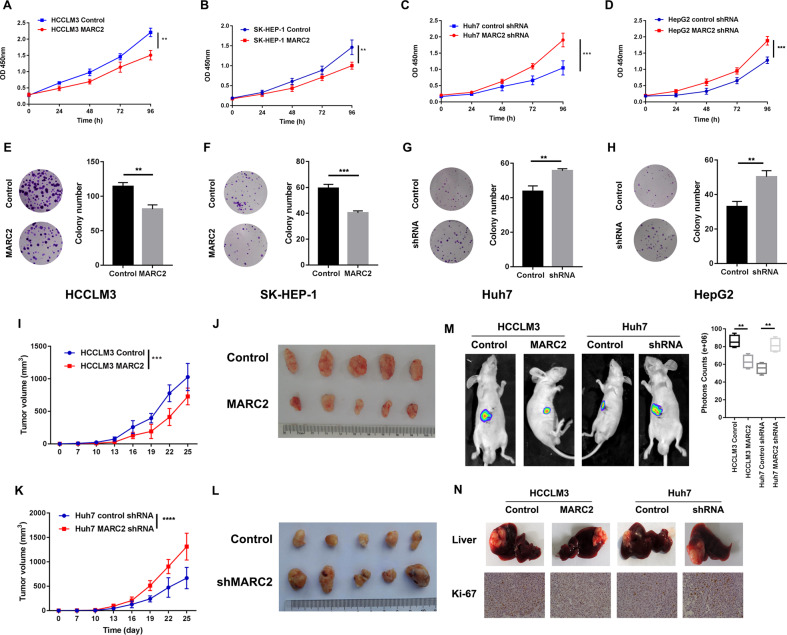
MARC2 suppresses the progress of HCC both in vitro and in vivo. **a**–**d** Proliferation rate was analyzed by CCK8 assay in the indicated HCC cell lines. **e**–**h** Images represented the colony formation assay in the indicated cell lines in the left panel and statistical analysis of colony numbers are in the right panel. **k**, **i** Tumor growth curves of subcutaneous tumor for the indicated cell lines. The tumor volume was measured per 3 day since the 7th day. **j**, **l** Representative images of subcutaneous xenograft derived from indicated cell lines. Five mice were used for each group. **m** Representative images of liver xenograft derived from indicated cell lines in left panel; the statistical analysis of bioluminescence signal in the right panel. Four mice were used for each group. **n** Representative images of livers (up) and IHC staining of Ki-67 (down) for the liver xenograft models derived from the indicated cell lines. The data are presented as mean ± SD. *p < 0.05; **p < 0.01; ***p < 0.001, all of which were considered statistically significant.

To determine the impact of MARC2 on HCC, its mRNA and protein expression levels were evaluated in normal (THLE-3) and different HCC cell lines. The majority of the HCC cell lines exhibited a relatively lower expression of MARC2 compared with that noted for THLE-3 (Fig. S2A, B). Subsequently, stable-transfected cell lines were established by overexpressing MARC2 in HCCLM3 and SK-HEP-1 cells which had a relatively lower expression of MARC2. In addition, silencing of MARC2 was performed in Huh7 and HepG2 cell lines that exhibited a relatively higher expression of MARC2. The efficiency of lentiviral (Lv) transfection was assessed by real-time PCR assay, and Lv-shRNA-1 (63%) was selected as the best candidate for silencing MARC2 expression (Fig. S2C, D). The proliferation was measured by the CCK8 and colony formation assays. The CCK8 assay results indicated that MARC2 overexpression inhibited the proliferation of HCC cells (Fig. 2a, b), whereas knockdown of MARC2 significantly increased cell growth (Fig. 2c, d). The colony formation results indicated that overexpression of MARC2 decreased the colony numbers of both HCCLM3 and SK-HEP-1 cell lines (Fig. 2e, f). While downregulation MARC2 promoted colony formation (Fig. 2g, h). The results of in vivo experiments were consistent with those of in vitro studies. Overexpression of MARC2 decreased the tumorigenicity to a certain extent, while the knockdown of MARC2 resulted in larger tumor volumes and enhanced growth rates (Fig. 2i–l). Overexpression of MARC2 inhibited tumor growth in the xenograft model, while its knockdown increased the growth rate of the xenograft tumors (Fig. 2m). The IHC staining results indicated that xenograft tumors in the MARC2 overexpression group exhibited a weaker staining of Ki-67 compared with those of the control group, whereas stronger staining was obtained for the MARC2 knockdown group (Fig. 2n).

### MARC2 suppresses the progression of HCC by regulating the protein expression of p27

**Fig. 3:**
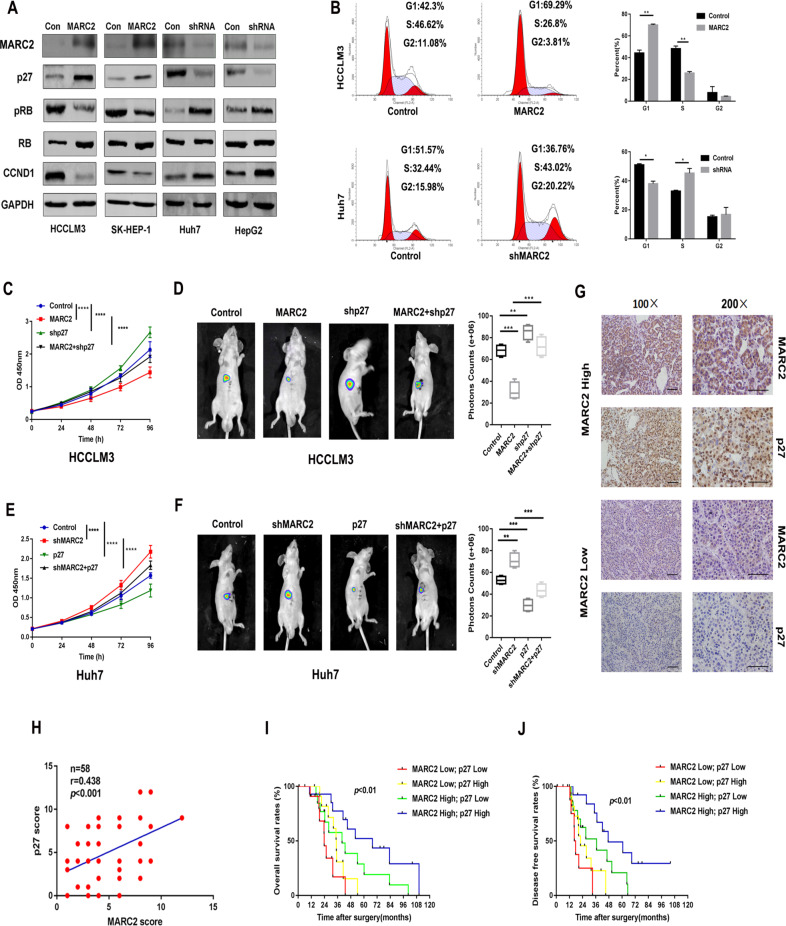
MARC2 suppresses the progression of HCC via regulating the protein expression of p27. **a** Expression of MARC2 and cell cycle associated signal proteins in the indicated cell lines was measured by western blot. **b** Representative images of cell cycle phase in the indicated cell lines in the left panel and statistical analysis in the right panel. **c**, **e** The growth curves of the indicated cell lines representing the proliferation rates were measured by CCK8 assay. **d**, **f** Representative images of liver xenograft derived from indicated cell lines in the left panel and the statistical analysis of bioluminescence signal in the right panel. **c**, **d** HCCLM3 cell line was used for the cell and mice experiment. **e**, **f** Huh7 cell line was used for the cell and mice experiment. **g** IHC staining of MARC2 and p27 in human HCC tissues. Bar = 100 µm (*100); Bar = 50 µm (*200). **h** IHC scores were used to exam correlation of protein expression between p27 and MARC2. The Pearson correlation coefficient was used to determine the correlation. **i**, **j** The Kaplan–Meier method was used to determine the overall survival and disease free survival of patient with HCC; the log-rank test was used to compare survival between different groups. *p < 0.05; **p < 0.01; ***p < 0.001, all of which were considered statistically significant.

The present work based on the framework of the TCGA PanCancer Atlas revealed that genetic alterations in signaling pathways that control cell cycle was a common hallmark of HCC [12]. We found MARC2 overexpression increased the accumulation of p27 and decreased the expression levels of pRB and CCND1. And knockdown of MARC2 decreased p27 and increased pRB and CCND1 levels (Fig. 3a). However, MARC2 did not influence the gene expression level of the p27 (Fig. S3A, B). Flow cytometer analysis indicated that increased MARC2 levels induced cell cycle arrest at the G1 phase, whereas knockdown of MARC2 facilitated cells passing through the G1/S checkpoint (Fig. 3b). Knockdown of p27 in HCCLM3 cells that overexpressed MARC2 inhibited the tumor suppressor effect of MARC2 both in vitro and in vivo (Fig. 3c, d). Overexpression of p27 in shMARC2-Huh7 cells could also inhibit shMARC2-induced HCC progression (Fig. 3e, f). The knockdown and overexpression efficiency of p27 in HCCLM3 and Huh7 cell lines had been measured by qPCR (Fig. S3C, D). IHC staining indicated a positive correlation between the protein expression of MARC2 and p27 in human HCC tissues (Fig. 3g, h). Moreover, patients with MARC2 and p27 co-expression exhibited considerably better prognosis (Fig. 3i, j).

### MARC2 regulates the expression of p27 via the Hippo signaling pathway

**Fig. 4:**
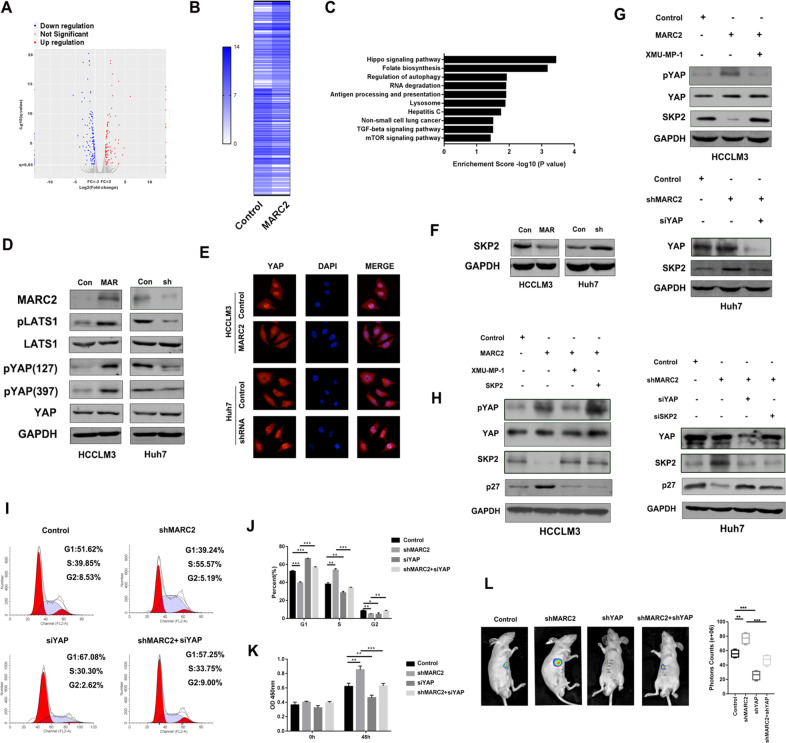
MARC2 regulates the expression of p27 via Hippo signaling pathway. **a** Volcano plot for different expression genes between HCCLM3 control and HCCLM3-MARC2 cell lines. Red plots represented the upregulated genes and blue plots represented the downregulated genes. The significant threshold were |fold change| > 2 and *p* < 0.05. **b** Heatmap representing the mRNA expression of significant different expression genes between HCCLM3 control and HCCLM3-MARC2 cells. **c** Significant different expression genes were filtered and enriched in KEGG signaling pathway; the Top10 signaling pathways were listed and ranked by *P* value. **d** Representative the WB images for Hippo signaling pathway in indicated cell lines. Con: Control; MAR: MARC2 overexpression; sh: MARC2 shRNA. **e** Expression of YAP in the indicated cells was examined by immunofluorescence. Single and merged images were taken in the indicated cells to show immunofluorescence staining of YAP (red), accompanied by the cell nucleus (blue) stained by DAPI. **f** Expression of SKP2 was detected by western blot in indicated cell lines. **g** XMU-MP-1 was used to active the YAP. 3 μM XMU-MP-1 was added to MARC2-HCCLM3 cell lines, and the expression of pYAP, YAP, and SKP2 was detected by western blot in the upper panel. SiYAP was used in shMARC2-Huh7 cell lines to suppress the Hippo signaling pathway, and the expression of YAP and SKP2 was detected by western blot in the lower panel. **h** To further confirmed that MARC2 regulated p27 via YAP/SKP2 axis, 3 μM XMU-MP-1 and SKP2 overexpression plasmid were added to MARC2-HCCLM3 cell lines respectively, and the expression of pYAP, YAP, SKP2, and p27 was detected by western blot in the left panel. SiYAP and siSKP2 were added to shMARC2-Huh7 cell lines respectively, and the expression of YAP, SKP2, and p27 was detected by western blot in the right panel. **i** Representative images for cell cycle assays in indicated cell lines. **j** Statistical analysis of cell cycle. **k** Proliferation rates were measured by CCK8 assay in the indicated cell lines at 0 h and 48 h. **l** Representative images of liver xenograft derived from indicated cell lines in the left panel; the statistical analysis of bioluminescence signal in the right panel. **i**–**l** Huh7 cell line was used for the experiment. *p < 0.05; **p < 0.01; ***p < 0.001, all of which were considered statistically significant.

To further explore the mechanism of MARC2 regulating p27, mRNA sequencing analysis was performed. The differential expression genes (|fold change| > 2, *p* < 0.05) were filtered and enriched in the signaling pathway (Fig. 4a, b). The results indicated that MARC2 could regulate the Hippo signaling pathway (Fig. 4c). The protein expression levels of key effectors of Hippo signaling pathway were evaluated using WB. The upregulated MARC2 resulted in increasing expression of pLATS1 and pYAP, while downregulated MARC2 got the opposite results (Fig. 4d). Phosphorylated YAP could prevent its nuclear translocation and its carcinogenic effect [13–14]. The IF results indicated that MARC2 overexpression inhibited YAP nuclear translocation, whereas decreased MARC2 expression promoted YAP nuclear translocation (Fig. 4e). A recent study demonstrated that inactivated YAP, which was the key effector of the Hippo signaling pathway, could directly suppress the expression of SKP2 and induce cell cycle arrest by causing the accumulating of p27 [15]. Therefore, we hypothesized that MARC2 could regulate cell cycle via the YAP/SKP2 axis. The WB result revealed that elevated MARC2 suppressed expression of SKP2, whereas downregulated MARC2 increased expression of SKP2 (Fig. 4f). The activation of YAP using XMU-MP-1 in MARC2-HCCLM3 cells attenuated the downregulation of SKP2 expression and the knockdown of YAP in shMARC2-Huh7 cells attenuated the upregulation of SKP2 induced by shMARC2 (Fig. 4g). The efficiency of siRNA-YAP was measured by qPCR (Fig. S3E).

Subsequently, we evaluated whether the YAP/SKP2 axis was required for MARC2-mediated expression of p27. The inactivation of the Hippo signaling pathway using XMU-MP-1 or overexpression of SKP2 in MARC2-HCCLM3 cell lines could attenuate the expression levels of p27, which were elevated by MARC2 overexpression (Fig. 4h). The knockdown of YAP or SKP2 in shMARC2-Huh7 cell lines could rescue the expression levels of p27 that were downergulated by shMARC2 (Fig. 4h). The efficiencies of silencing SKP2 were measured by qPCR (Fig. S3F). These data indicated that MARC2 regulated p27 via the Hippo signaling pathway. Knockdown of YAP by siRNA also abrogated the effects of shMARC2 on the cell cycle (Fig. 4i, j). The CCK8 assay results revealed that knockdown of YAP attenuated the effects of shMARC2 on cell proliferation (Fig. 4k). In vivo experiments further demonstrated that downregulation of YAP prevented the progression of HCC induced by shMARC2 (Fig. 4l). These results revealed that the Hippo signaling pathway was indispensable for downregulating MARC2-mediated progression of HCC.

### MARC2 regulates HNF4A by formation of a feedback loop via the Hippo signaling pathway

**Fig. 5:**
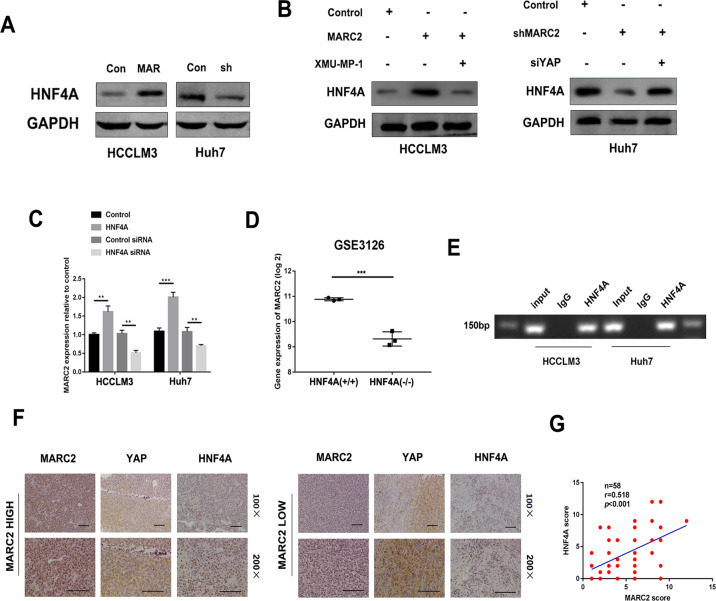
MARC2 regulates HNF4A by formation of a feedback loop via the Hippo signaling pathway. **a, b** Protein expression of HNF4A was detected by western blot in indicated cell lines. XMU-MP-1 was used to active the YAP, while the siYAP was used to suppress the Hippo signaling pathway. To further confirmed regulated HNF4A via Hippo signaling pathway, XMU-MP-1 was added to HCCLM3-MARC2 cell line and siYAP was added to Huh7-shMARC2 cell line. **c** Gene expression of MARC2 was measured by real-time PCR in indicated cell lines. **d** Gene expression of MARC2 in HNF4A KO mice. Data were obtained from GSE3126. HNF4A (+/+) was used as control group and HNF4A (−/−) represented the HNF4A knockout group. **e** CHIP-PCR assay showed HNF4A was recruited to promoter region of MARC2. **f** Representative the images of IHC staining for MARC2, YAP, and HNF4A in human HCC tissues. Bar = 100 µm (*100); Bar = 50 µm (*200). **g** IHC scores were used to exam correlation of protein expression of HNF4A and MARC2. The Pearson correlation coefficient was used to determine the relationship of protein expression between HNF4A and MARC2. *p < 0.05; **p < 0.01; ***p < 0.001.

HNF4A is a key downstream transcription factor of the Hippo signaling pathway [16]. The results revealed that overexpression of MARC2 increased the expression of HNF4A, whereas its downergulation suppressed the expression of HNF4A (Fig. 5a). The activation of YAP using XMU-MP-1 in MARC2-HCCLM3 cells attenuated the upregulation of HNF4A expression that was induced by MARC2 while the knockdown of YAP in shMARC2-Huh7 cells rescued the downregulation of HNF4A induced by shMARC2 (Fig. 5b). These data indicated that MARC2 regulated HNF4A via the Hippo signaling pathway. Interestingly, we found overexpression of HNF4A also increased the expression of MARC2, whereas knockdown of HNF4A suppressed the expression of MARC2 (Fig. 5c, Fig. S3G, H). Transgenic knockout HNF4A in mice could also decrease the expression of MARC2 (Fig. 5d). Chip-qPCR assays indicated that HNF4A was recruited to the promoter of MARC2 in both HCCLM3 and Huh7 cell lines (Fig. 5e). The IHC results showed that the protein expression levels of HNF4A exhibited a positive correlation with MARC2 in HCC tissues (Fig. 5f, g). These results revealed that MARC2 regulated HNF4A by formation of a feedback loop via Hippo signaling pathway and had a close relationship with HNF4A in HCC tissues.

### MARC2 combines with RNF123 to prevent the degradation of p27

**Fig. 6:**
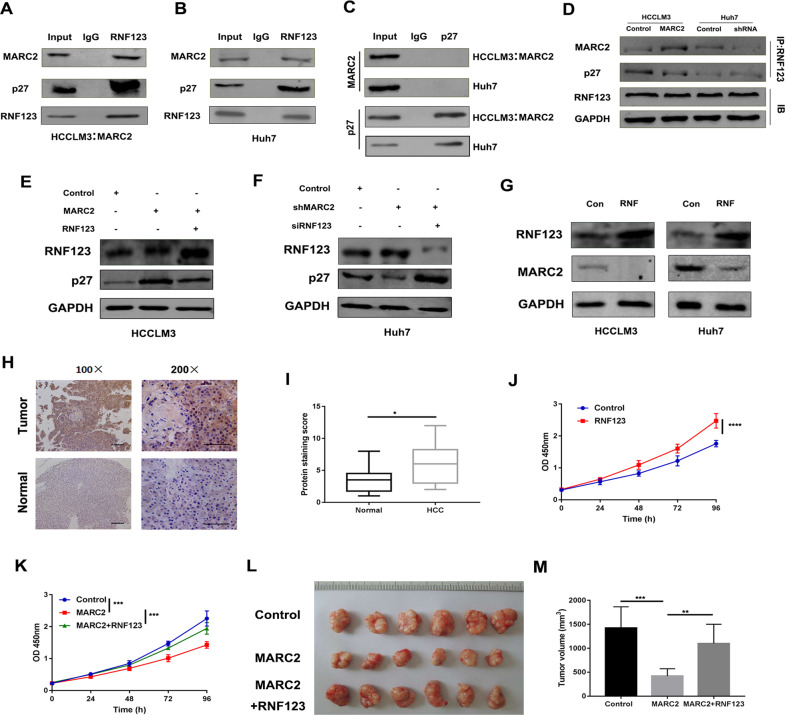
MARC2 combines with RNF123 to prevent the degradation of p27. **a**, **b** The indicated cell lysates were prepared and immunoprecipitated with anti-RNF123 antibody. The protein expressions of MARC2, p27 and RNF123 were detected by western blot. HCCLM3 with overexpression of MARC2 represented the exogenous MARC2 interaction with RNF123, and Huh7 cell line represented the endogenous MARC2 interaction with RNF123. **c** The indicated cell lysates were prepared and immunoprecipitated with anti-p27 antibody. Immunoprecipitates and cell lysates were analyzed by western blot. **d** The indicated cell lysates were prepared and immunoprecipitated with anti-RNF123 antibody. The protein expressions of MARC2, p27, and RNF123 were detected by western blot. **e**, **f** The protein expression of RNF123 and p27 was detected by western blot in the indicated cell lines. **g** The protein expression of RNF123 and MARC2 was detected by western blot in the indicated cell lines. Con: Control; RNF: RNF123 overexpression. **h** Representative images of IHC staining for RNF123 in human HCC and adjacent normal tissues. Bar = 100 µm (*100); Bar = 50 µm (*200). **i** RNF123 IHC scores were used to compare the expression of RNF123 between normal tissues and HCC tissues. **j**, **k** Proliferation rate was analyzed by CCK8 assay in the indicated cell lines. **l** The images of subcutaneous xenograft derived from indicated cell lines. **m** Statistical analysis of tumor volume. **j**–**m** The experiment was derived from HCCLM3 cell line. *p < 0.05; **p < 0.01; ***p < 0.001.

RNF123 is also known as KPC1 and is an E3 ubiquitin ligase that can regulate the proteolysis of p27 in an SKP2-independent way during the cell cycle [17, 18]. A mass spectrometry study demonstrated that RNF123 could combine with MARC2 in HEK293T cells [19]. Therefore, we investigated whether MARC2 could regulate the expression levels of p27 via RNF123 in HCC. Co-IP was performed and the data indicated that RNF123 was interacted with either exogenous or endogenous MARC2 (Fig. 6a, b). Immunofluorescence staining revealed cytoplasm colocalization of MARC2 and RNF123 (Fig. S4A). However, no direct interaction was noted between p27 and MARC2 (Fig. 6c). We further tested whether MARC2 could prevent the degradation of p27 by competitive binding to RNF123. To confirm this effect, the cell lysates of MARC2-HCCLM3 and shMARC2-Huh7 cell lines were immunoprecipitated using antibodies against RNF123. The results revealed that overexpression of MARC2 reduced the interaction between RNF123 and p27, while the knockdown of MARC2 promoted this interaction (Fig. 6d). In addition, the overexpression or the knockdown of MARC2 did not affect the expression of RNF123 (Fig. 6d). Overexpression of RNF123 in MARC2-HCCLM3 cells attenuated the expression of p27 (Fig. 6e). Downregulation of RNF123 in shMARC2-Huh7 cells restored the expression of p27 (Fig. 6f). The combination of MARC2 and RNF123 could increase the degradation of MARC2 (Fig. 6g). The expression levels of RNF123 were higher in HCC tissues compared with those noted in the normal tissues (Fig. 6h, i). Overexpression of RNF123 enhanced the proliferation of HCC cells (Fig. 6j). In addition, overexpression of RNF123 could also abrogate the effects of MARC2 on HCC cells both in vitro and in vivo (Fig. 6k–m).

### MARC2 is downregulated by methylation

**Fig. 7:**
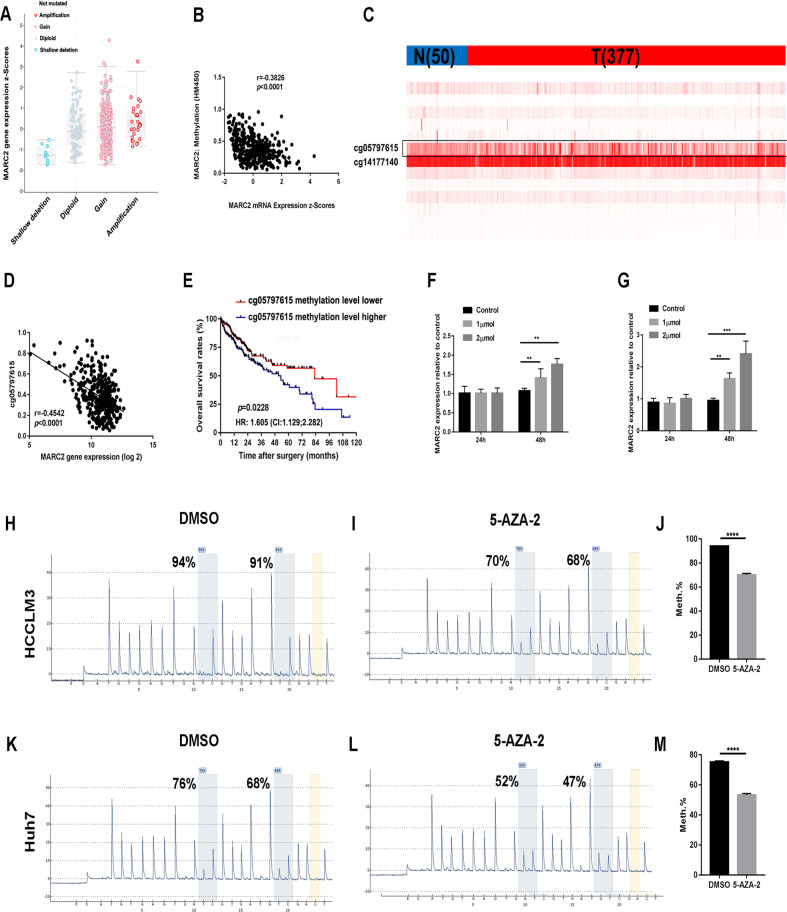
MARC2 is downregulated by methylation. **a** Gene expression of MARC2 in different copy number groups. Shallow deletion: 0.5; Diploid: 1; Gain: 1.5; Amplification: 2. **b** Correlation between MARC2 gene expression and methylation score of MARC2. The Pearson correlation coefficient was used to determine the relationship. **c** Heatmap for the CpG sites of MARC2. N represented the normal tissue (*n* = 50), and T represented the tumor tissue (*n* = 377). **d** Correlation between MARC2 gene expression and methylation score of cg05797615. The Pearson correlation coefficient was used to determine the relationship. **e** The Kaplan–Meier method was used to compare the OS between the patient group with high methylation score of cg05797615 and patient group with low methylation score of cg05797615. **f**, **g** 0, 1, 2 µmol 5-Aza-2′-deoxycytidine was added to HCCLM3 and Huh7 cell lines respectively. qPCR was used to analyze the gene expression of MARC2 in HCCLM3 and Huh7 cell lines treated with 5-Aza-2′-deoxycytidine at 24 h and 48 h. **h**–**m** Pyrosequencing was used to analyze the methylation level of cg05797615 in HCCLM3 and Huh7 cell lines treated with 5-Aza-2′-deoxycytidine in the left panel and statistical analysis in the right panel. *p < 0.05; **p < 0.01; ***p < 0.001.

MARC2 is frequently downregulated in HCC tissues (Fig. 1c). The mechanism of this process is unknown. The copy number of MARC2 were increased in 8% of the patients (29/361) and a shallow deletion was noted in 3% of the patients (10/361) (Fig. 7a). Gene expression of MARC2 was slightly associated with the copy number, indicating the presence of other confounding factors that could influence expression levels. The Infinium Human Methylation 450 Beadchip data and the mRNA expression data of MARC2 from the TCGA were combined to assess the association of MARC2 expression and its DNA methylation. The results indicated that the mRNA expression levels of MARC2 were negatively associated with its DNA methylation (Fig. 7b). Two CpG sites (cg05797615 and cg14177140) were significantly hypermethylated and the methylation score of cg05797615 varied in HCC tissues (Fig. 7c). The regression analysis indicated a negative correlation between the methylation levels of cg05797615 and MARC2 expression (Fig. 7d). Kaplan–Meier analysis further demonstrated that the patients with high methylation levels of cg05797615 exhibited significantly shorter OS compared with that of the low level group (Fig. 7e). However, the same results were not noted on cg14177140 (Fig. S4B, C), indicating that the methylation status of the CpG site cg05797615 played a key role in regulating the expression of MARC2. To further confirm that MARC2 was suppressed by methylation, we treated HCCLM3 and Huh7 cells with 5-Aza-2′-deoxycytidine. The results revealed that 5-Aza-2′-deoxycytidine increased the expression levels of MARC2 in both a dose- and time-dependent way (Fig. 7f, g). Pyrosequencing results indicated that 5-Aza-2′-deoxycytidine significantly decreased the methylation levels of cg05797615 in HCCLM3 and Huh7 cells (Fig. 7h–m). These data indicated that MARC2 was suppressed by methylation in HCC.

## Discussion

In the present study, we demonstrated that metabolic dysfunction could be served as a key driver for the development of HCC. MARC2 was one of the dysregulated metabolism enzymes and its expression was downregulated in HCC. The expression of MARC2 was consistent with the progression of HCC development. The expression of MARC2 was an independent favorable indicator for both OS and DFS of HCC. Our loss-of-function and gain-of-function experiments revealed that MARC2 overexpression and knockdown attenuated and promoted HCC proliferation respectively. These findings were noted both in vitro and in vivo. Therefore, MARC2 is a candidate tumor suppressor for HCC risk prognosis and therapy.

p27 is a cyclin-dependent kinase inhibitor that controls the cell-cycle progression at the G1 phase. The degradation of p27 is required for the cellular transition from quiescence to the proliferative status. Our results revealed that p27 was required for MARC2-mediated tumor suppression. We observed a positive correlation between MARC2 and p27 in HCC tissues and the combination of MARC2 and p27 expression levels exhibited a higher prognostic value than either factor alone. YAP and RNF123 were required for this process. MARC2 competed with RNF123 for preventing the degradation of p27. The direct mechanism of YAP regulation by MARC2 is still unknown. A recent study indicated that MARC2 affected the ATP production pathway and that it may play a role in energy homeostasis [20]. We speculated that the activation of the Hippo signaling pathway caused by the downregulation of MARC2 might be due to the energy metabolic stress.

The dysregulation of MARC2 was significantly associated with the methylation status of the MARC2 promoter, notably the CpG site cg05797615. qPCR and pyrosequencing results further confirmed that MARC2 could be regulated by epigenetic modification.

In a conclusion, the present study demonstrated that MARC2 expression was downregulated by methylation in HCC cells. Downregulation of MARC2 expression promoted the progression of HCC both in vitro and in vivo by mediating the expression of p27 via the Hippo signaling pathway and RNF123. MARC2 regulated HNF4A via the Hippo signaling pathway and HNF4A was recruited to the promoter region of MARC2 causing an increase in its expression and forming a feedback loop (Fig. S5). The present study indicated a central role of MARC2 in HCC progression and the data suggest that MARC2 could be a useful biomarker for HCC prognosis.

## Materials and methods

### TCGA-HCC data and GEO-HCC data

TCGA-HCC data were obtained from Cbioportal (https://www.cbioportal.org). The GEO data were obtained from oncomine (https://www.oncomine.org/). Data were secondary analyzed by HCCDB (http://lifeome.net/database/hccdb/home.html) and GraphPad Prism 7.0.

### Co-Immunoprecipitation (co-IP) and Western blot (WB)

Cells were incubated with pre-cold IP lysis buffer for 5 min and then transferred to a 1.5 mL tube for centrifuged at 13,000 *g* for 10 min. The supernatant was collected and completed the IP process following the protocol of A Dynabeads protein G IP kit (Thermofisher, US). The WB was performed as previously described [21]. MARC2 (#ab224097), p27 (#ab32034), CCND1 (#ab16663) and SKP2 (#ab183039) were purchased from Abcam; YAP (#14074S), HNF4A (#3113S), pRB (#9301T), pLATS1 (#9157), LATS1 (#9153), pYAP(127) (#13008) and pYAP(397) (#13619) were purchased from Cell Signaling Technology; RNF123 (#sc-101122) used for co-IP and RB (#sc-74562) was purchased from Santa [22, 23].

### Statistical analysis

The R studio and Graphpad Prism 7 were used for the statistical analysis. The statistical analysis for different group and gene expression in databases was performed by using the two-tailed Student’s *t* test or one-way ANOVA. The results were presented as means ± SD. Overall survival (OS) and DFS were compared by the Kaplan–Meier method, and significance was determined by a Log-rank test. Associations between MARC2 expression and various clinic-pathological characteristics were assessed by *χ*2-tests. Cox’s proportional hazards regression model was used to identify independent prognostic factors. The significant threshold was defined as *p* < 0.05.

## Supplementary information

Supplemental manuscript

Supplemental Figure 1

Supplemental Figure 2

Supplemental Figure 3

Supplemental Figure 4

Supplemental Figure 5

## References

[1] Siegel RL, Miller KD, Jemal A (2020). Cancer statistics, 2020. CA Cancer J Clin.

[2] Massarweh NN, El-Serag HB (2017). Epidemiology of hepatocellular carcinoma and intrahepatic cholangiocarcinoma. Cancer Control.

[3] Guichard C, Amaddeo G, Imbeaud S, Ladeiro Y, Pelletier L, Maad IB (2012). Integrated analysis of somatic mutations and focal copy-number changes identifies key genes and pathways in hepatocellular carcinoma. Nat Genet.

[4] Wang X, Chen D, Chen B. The long-to-short-axis ratio and multifocality are associated with TP53 mutation status in surgically resected hepatocellular carcinomas. Acad Radiol. 2018; e-pub ahead of print 27 June 2018; 10.1016/j.acra.2018.04.021. 10.1016/j.acra.2018.04.021 29941397

[5] Wahl B, Reichmann D, Niks D, Krompholz N, Havemeyer A, Clement B (2010). Biochemical and spectroscopic characterization of the human mitochondrial amidoxime reducing components hmARC-1 and hmARC-2 suggests the existence of a new molybdenum enzyme family in eukaryotes. J Biol Chem.

[6] Plitzko B, Ott G, Reichmann D, Henderson CJ, Wolf CR, Mendel R (2013). The involvement of mitochondrial amidoxime reducing components 1 and 2 and mitochondrial cytochrome b5 in N-reductive metabolism in human cells. J Biol Chem.

[7] Sparacino-Watkins CE, Tejero J, Sun B, Gauthier MC, Thomas J, Ragireddy V (2014). Nitrite reductase and nitric-oxide synthase activity of the mitochondrial molybdopterin enzymes mARC1 and mARC2. J Biol Chem.

[8] Kotthaus J, Wahl B, Havemeyer A, Kotthaus J, Schade D, Garbe-Schonberg D (2011). Reduction of N(omega)-hydroxy-L-arginine by the mitochondrial amidoxime reducing component (mARC). Biochem J.

[9] Neve EP, Nordling A, Andersson TB, Hellman U, Diczfalusy U, Johansson I (2012). Amidoxime reductase system containing cytochrome b5 type B (CYB5B) and MOSC2 is of importance for lipid synthesis in adipocyte mitochondria. J Biol Chem.

[10] Li Z, Zhang Y, Zhang Z, Zhao Z, Lv Q (2019). A four-gene signature predicts the efficacy of paclitaxel-based neoadjuvant therapy in human epidermal growth factor receptor 2-negative breast cancer. J Cell Biochem.

[11] Mikula M, Rubel T, Karczmarski J, Goryca K, Dadlez M, Ostrowski J (2011). Integrating proteomic and transcriptomic high-throughput surveys for search of new biomarkers of colon tumors. Funct Integr Genom.

[12] Sanchez-Vega F, Mina M, Armenia J, Chatila WK, Luna A, La KC (2018). Oncogenic signaling pathways in the cancer genome atlas. Cell.

[13] Yu FX, Guan KL (2013). The Hippo pathway: regulators and regulations. Genes Dev.

[14] Mo JS, Park HW, Guan KL (2014). The Hippo signaling pathway in stem cell biology and cancer. EMBO Rep..

[15] Ganem NJ, Cornils H, Chiu SY, O’Rourke KP, Arnaud J, Yimlamai D (2014). Cytokinesis failure triggers hippo tumor suppressor pathway activation. Cell.

[16] Fitamant J, Kottakis F, Benhamouche S, Tian HS, Chuvin N, Parachoniak CA (2015). YAP inhibition restores hepatocyte differentiation in advanced HCC, leading to tumor regression. Cell Rep..

[17] Kamura T, Hara T, Matsumoto M, Ishida N, Okumura F, Hatakeyama S (2004). Cytoplasmic ubiquitin ligase KPC regulates proteolysis of p27(Kip1) at G1 phase. Nat Cell Biol.

[18] Dobashi Y, Tsubochi H, Minegishi K, Kitagawa M, Otani S, Ooi A (2017). Regulation of p27 by ubiquitin ligases and its pathological significance in human lung carcinomas. Hum Pathol.

[19] Khanna R, Krishnamoorthy V, Parnaik VK (2018). E3 ubiquitin ligase RNF123 targets lamin B1 and lamin-binding proteins. FEBS J.

[20] Rixen S, Havemeyer A, Tyl-Bielicka A, Pysniak K, Gajewska M, Kulecka M (2019). Mitochondrial amidoxime-reducing component 2 (MARC2) has a significant role in N-reductive activity and energy metabolism. J Biol Chem.

[21] Liu Y, Meng F, Wang J, Liu M, Yang G, Song R (2019). A novel oxoglutarate dehydrogenase-like mediated miR-214/TWIST1 negative feedback loop inhibits pancreatic cancer growth and metastasis. Clin Cancer Res.

[22] Jang W, Kim T, Koo JS, Kim SK, Lim DS (2017). Mechanical cue-induced YAP instructs Skp2-dependent cell cycle exit and oncogenic signaling. EMBO J.

[23] Hnit SS, Xie C, Yao M, Holst J, Bensoussan A, De Souza P (2015). p27(Kip1) signaling: transcriptional and post-translational regulation. Int J Biochem Cell Biol.

