# Supplementary material for: A novel mitochondrial amidoxime reducing component 2 is a favorable indicator of cancer and suppresses the progression of hepatocellular carcinoma by regulating the expression of p27
Source: Oncogene. 2020 Aug 18;39(38):6099–112. doi: 10.1038/s41388-020-01417-6 (PMC7498369; doi:10.1038/s41388-020-01417-6)
Supplement: Supplementary file 1 — Supplemental manuscript [file 41388_2020_1417_MOESM1_ESM.docx]

**Supplementary materials and methods**

**HCC sample collection and cell lines**

The human HCC tissues and the adjacent normal tissues from 87 patients with HCC were collected at the First Affiliated Hospital of Harbin Medical University during surgical operation. The patient involved in this program did not received radiotherapy or chemotherapy before and after the surgery. All the patients had signed written informed consent and the Research Ethics Committee of the First Affiliated Hospital of Harbin Medical University approved the study. All the cell lines used in this study were purchased from Shanghai Cell Bank of Chinese Academy of Sciences (Shanghai, China). All the cell lines were authenticated by STR profiling and tested for mycoplasma contamination. All the cell lines were cultured in Dulbecco’s modified Eagle’s medium (DMEM) with 10% fetal bovine serum (FBS) and 1% antibiotics (100 U/ml penicillin and 100 μg/ml streptomycin). All cell lines were cultured at 37 °C in a 5% CO2 incubator.

**Immunohistochemical (IHC) staining**

Tissue sections deparaffinized were stained with diaminobenzidine (DAB kit; Vector Laboratories) and counterstained with hematoxylin (Sigma) to visualize the immunoreaction product following the protocol. Two independent pathologists from our hospital scored the tissue slides basing on both intensity and extent. The staining intensity was scored as 0 (negative), 1 (weak), 2 (moderate) and 3 (strong). The percentage scores were defined as: 0, less than 5%; 1, 5% to 25%; 2, 26% to 50%; 3, 51% to 75%; 4, more than 75%. The histologic score (H score) for each section was calculated with the following formula: histologic score = proportion score × intensity score. MARC2 (#ab224097), p27 (#ab32034) and KI-67 (#ab16667) were purchased from Abcam; YAP (#14074S) and HNF4A (#3113S) were purchased from Cell Signaling Technology; RNF123 (#NBP2-49467) was purchased from Novus Biologicals.

**Lentivirus, small interfering RNA and stable cell line construction.**

The lentiviral vector system (LV), short hairpin RNAs (LV-shRNA) and the empty vectors were purchased from GeneChem Corporation (Shanghai, China). Small interfering RNA (siRNA) was purchased from GenePharma Corporation (Suzhou, China). Stable cell lines expressing target gene or negative control was selected by adding 0.5 μg/ml puromycin into the medium.

**Quantitative real-time PCR (qPCR)**

Total RNA was extracted and quantified following the protocol of RNA purification Kits (Thermofisher, US). Quantitative real-time PCR was performed following the protocol of SYBR Green Power Master Mix (Promega, Madison, USA). Primers used for qRT-PCR experiments were listed

F: 5’-GAGAAATGGGAAGCAACAGA-3’,

R: 5’-GGCAGTTACACACAAACAGATTAC-3’ for MARC2;

F: 5’-AGAAGGCTGGGGCTCATTTG-3’,

R: 5’-AGGGGCCATCCACAGTCTTC-3’ for GAPDH.

**Cell proliferation and cell cycle assay**

Cells were seeded in 96 well plates with 2×10^3^ per well and cell viability was measured by Counting Kit-8 (CCK-8) assays (Dojindo Molecular Technologies, Inc., Japan) at different time points. For the colony formation, cells were seeded in a six well plate with 200 cells per well. Colonies were fixed by methanol and stained with crystal violet after 2 weeks. For cell cycle tests, 4×10^4^ cells were stained as the protocol of Cycle TESTTM PLUS DNA Reagent kit (BD Biosciences San Jose, CA) and were analyzed by flow cytometer (Beckman Coulter FC500).

***In vivo* tumor growth**

The Animal Ethics Committee of Harbin Medical University approved all experimental protocols involving animals. Four-six week old male BALB/c nude mice were purchased from the Shanghai Animal Centre (Shanghai, China). Flank subcutaneous xenografts were established by subcutaneous injection of 1×10^6^ cells suspended in 100μl PBS. After 6 weeks, subcutaneous tumors were removed and the volume was recorded by the formula: a×b^2^ (a: length, b: width). For the liver orthotropic xenograft implantation model, subcutaneous tumors were dissected into 1mm^3^ sections and the liver lobe for implantation was randomly selected. The location for tumor embedded was around the middle liver parenchyma of the liver lobe where was away from the edge. Tumor growth was monitored by bioluminescent signals twice a week. D-luciferin (Xenogen, Hopkinton, MA) was injected intraperitoneally into the mice at 100 mg per kilogram (animal weight), and bioluminescence was detected with Berthold NIGHTOWL LB983 imaging machine. Tumors were processed for IHC to assess proliferation.

**Immunofluorescence (IF) assays**

Cells were seeded on coverslips in six well plates for 24h. Then the cells were fixed with 4% paraformaldehyde and penetrated by 0.1% Triton-X-100. After incubating with primary antibody (YAP, #14074s, CST; MARC2, #sc-514202, Santa; RNF123, #NBP2-57940, Novus), second antibody (Invitrogen) and DAPI (Vector Laboratories) in sequence, the images of the cells were captured by DMRA fluorescence microscope (20×, Olympus).

**Chip-qPCR**

Cells were fixed by 37% formalehyde for 10min and neutralized by 1.25M glycine. Then the cells lysis was collected and sonication. The immunoprecipitation was performed using HNF4A antibody (#ab181604, Abcam). The PCR products were separated by agarose gel electrophoresis and visualized by ethilium bromide staining. The primers used for Chip-qPCR were listed.

MARC2 Forward: 5’ AACAAATCCATAAGTCGCAGC 3’

MARC2 Reverse: 5’ CCCACCCAGGAAAAAACC 3’

**Methylation**

Cells were seeded in a six well plate treated with 5-Aza-2'-deoxycytidine 0μmol, 1μmol, 2μmol for 24h and 48h, respectively. DMSO was used in the control group. For the Pyrosequencing, the total DNA was collected and purified for bisulfite conversion. The 60bp sequencing around cg05797615 was amplified and measured methylation level.

**Supplementary Figure legends**

**Figure S1.**

(A) MARC2 gene expression in different normal tissues. The data was derived from HCCDB. (B) MARC2 gene expression in different tumors. The data was derived from TCGA. (C) Valid gene expression of MARC2 in other GEO and CGC-LIRI datasets. The data was derived from HCCDB. (D) The Kaplan-Meier method was used to analyze the OS in GSE14520 and ICGC-LIRI-JP. The data was derived from HCCDB.

**Figure S2.**

(A) qPCR detected gene expression of MARC2 in different HCC cell lines. (B) Western blot detected protein expression of MARC2 in different HCC cell lines. (C-D) Efficiency of lenti-MARC2 and lenti-shRNA in cell lines was measured by real-time PCR. Figures represent gene expression of MARC2 in indicated cell lines.

**Figure S3**

(A and B) Gene expression of p27 in indicated cell lines. (C) Knockdown efficiency of p27 by shRNA was measured by qPCR in HCCLM3. (D) Overexpression efficiency of p27 in Huh7 was measured by qPCR. (E) The efficiency of knockdown YAP by siRNA in Huh7 cell line was measured by qPCR. (F) The efficiencies of knockdown SKP2 in Huh7 cell line were measured by qPCR. (G) The efficiency of overexpression HNF4A in HCCLM3 and Huh7 cell lines. (H) The efficiency of knockdown HNF4A in HCCLM3 and Huh7 cell lines.

**Figure S4.**

(A) Represented the images of IF for MARC2 and RNF123 in HCCLM3 and HCCLM3-MARC2 cell lines. (B) Correlation between MARC2 gene expression and methylation score of cg14177140. The Pearson correlation coefficient was used to determine the relationship. (C) The Kaplan-Meier method was used to compare the OS between the patient group with high methylation score of cg14177140 and patient group with low methylation score of cg14177140.

**Figure S5**

Summarize graph
