# Supplementary figures and images for: A novel mitochondrial amidoxime reducing component 2 is a favorable indicator of cancer and suppresses the progression of hepatocellular carcinoma by regulating the expression of p27
Source: Oncogene. 2020 Aug 18;39(38):6099–112. doi: 10.1038/s41388-020-01417-6 (PMC7498369; doi:10.1038/s41388-020-01417-6)

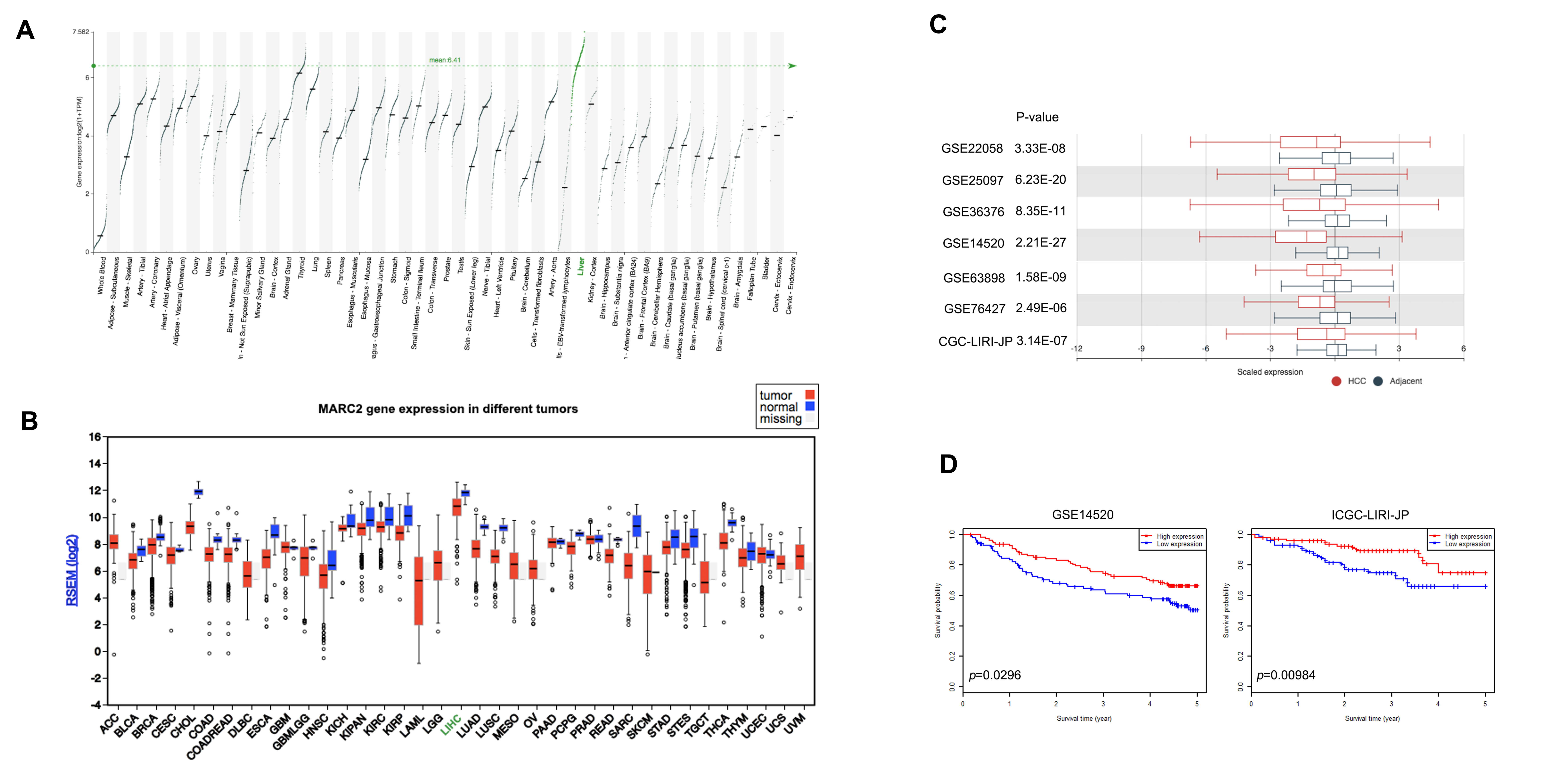

Supplement: Supplementary file 2 — Supplemental Figure 1 [file 41388_2020_1417_MOESM2_ESM.tif]

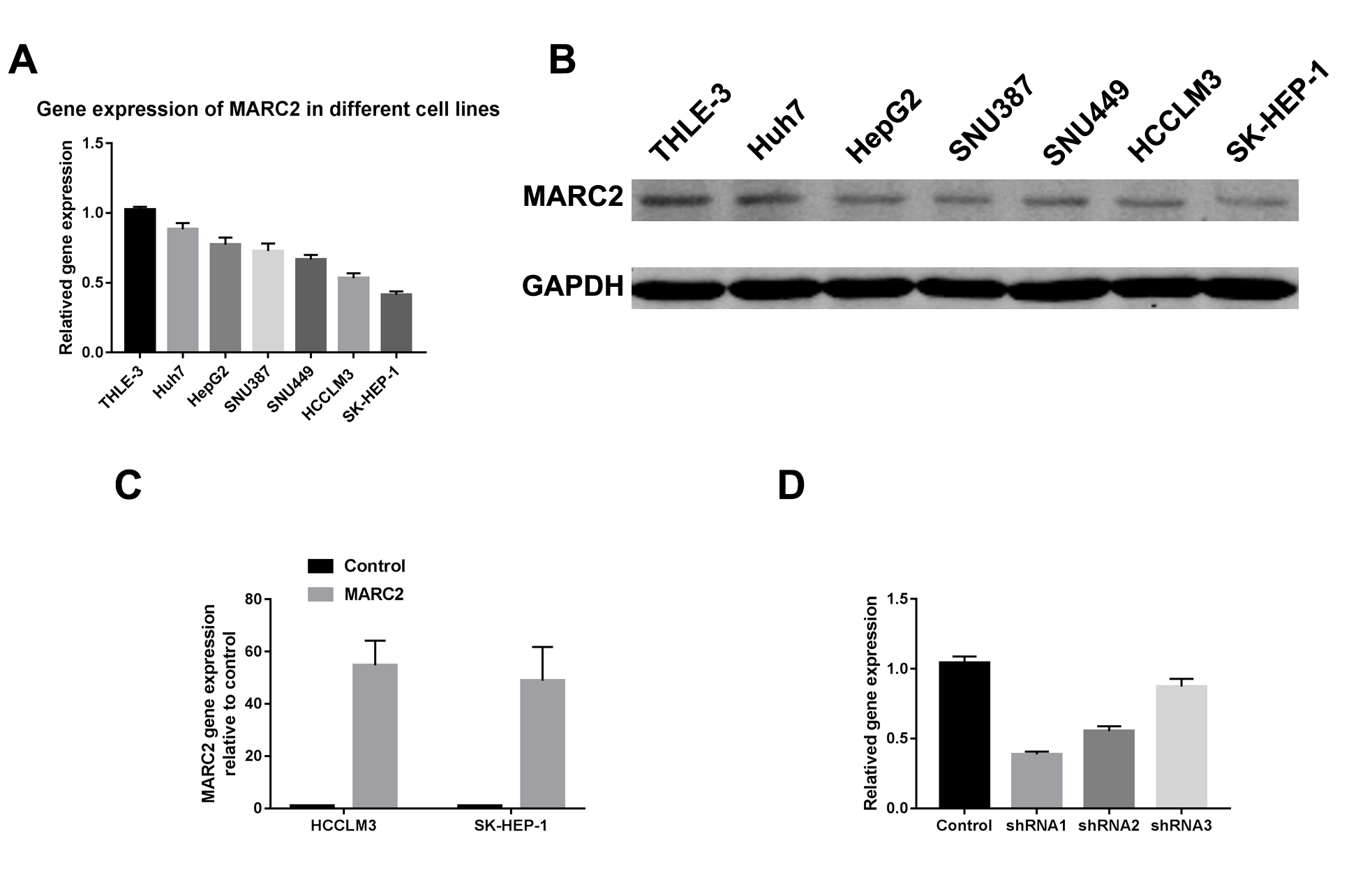

Supplement: Supplementary file 3 — Supplemental Figure 2 [file 41388_2020_1417_MOESM3_ESM.tif]

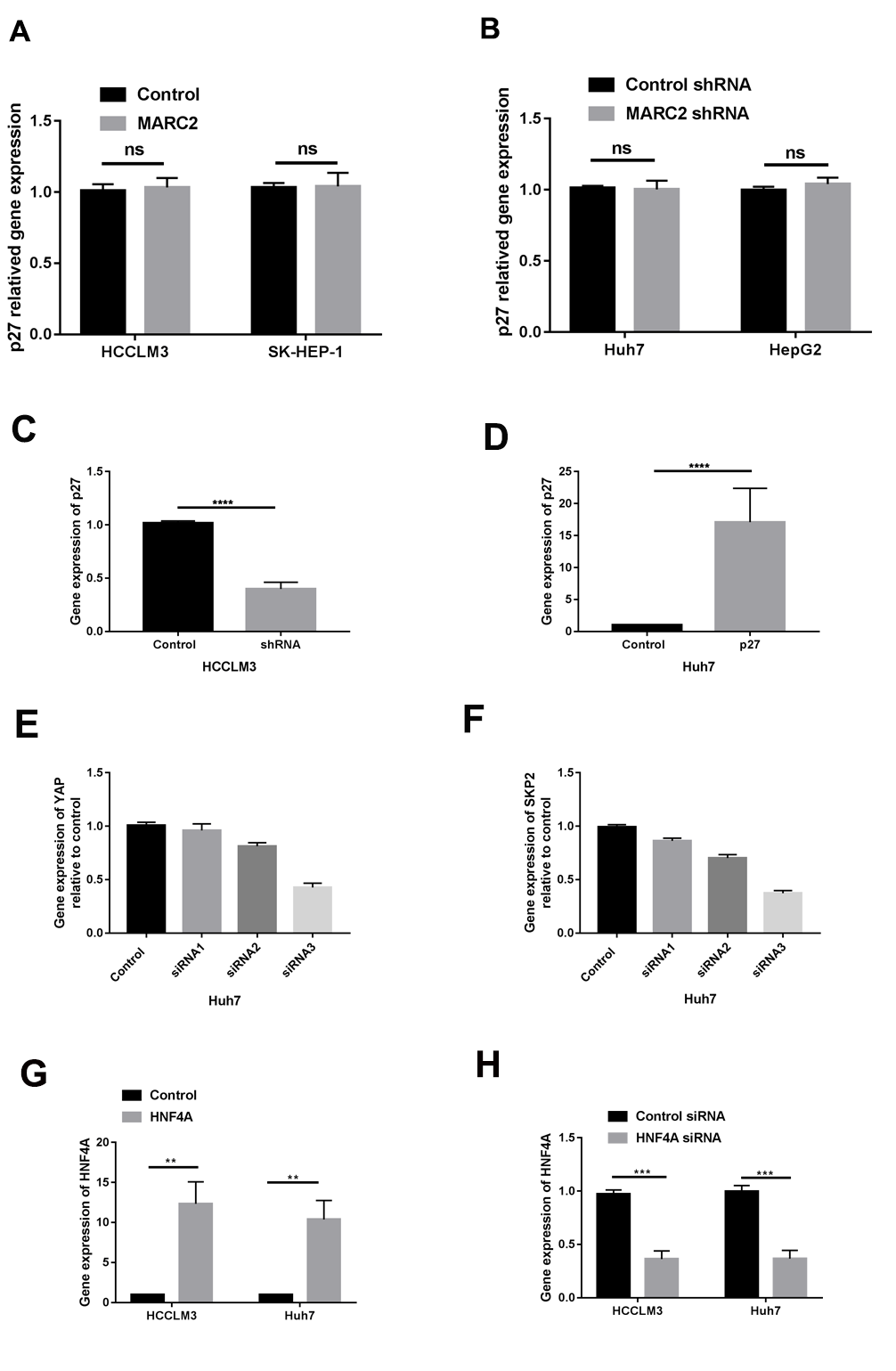

Supplement: Supplementary file 4 — Supplemental Figure 3 [file 41388_2020_1417_MOESM4_ESM.tif]

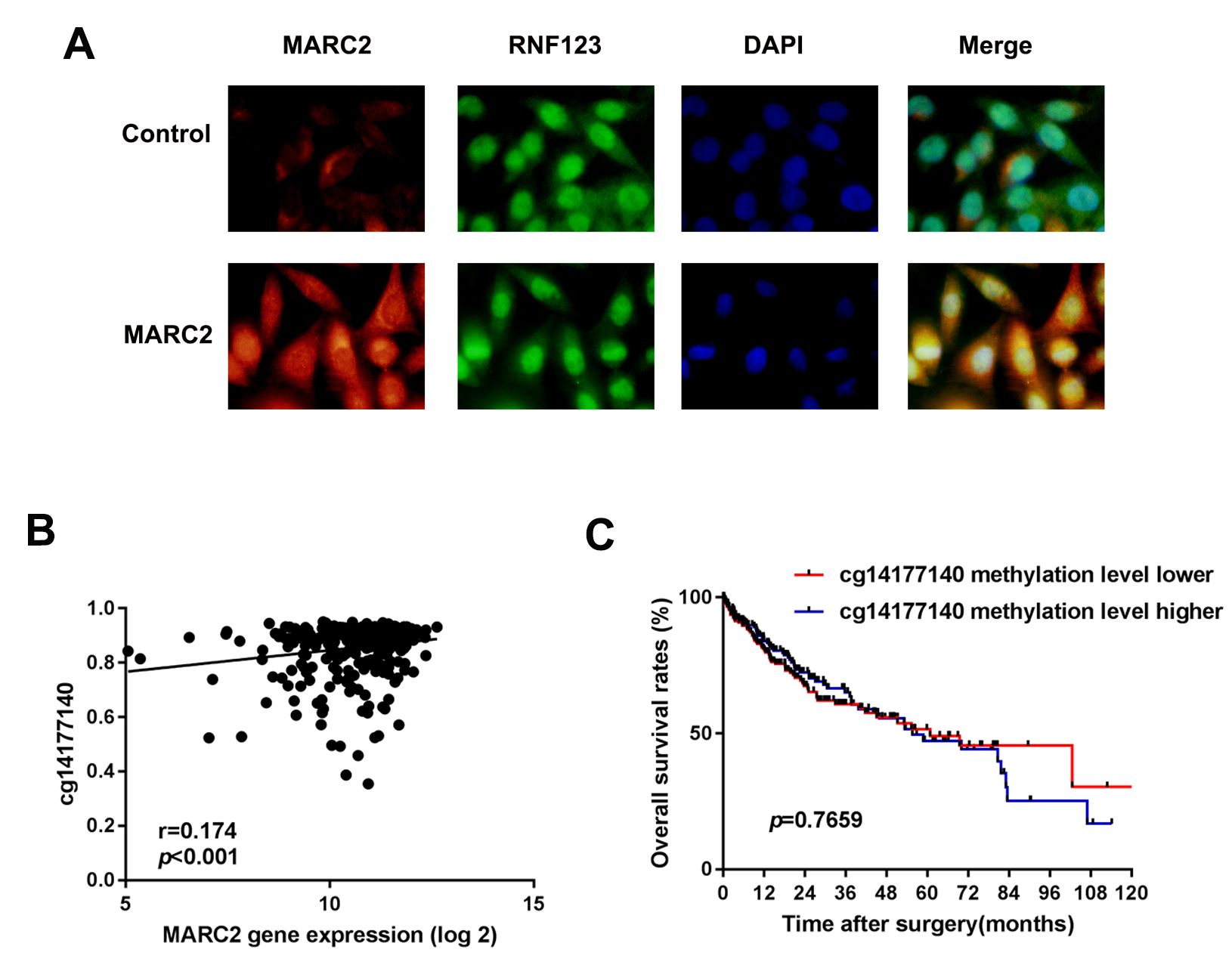

Supplement: Supplementary file 5 — Supplemental Figure 4 [file 41388_2020_1417_MOESM5_ESM.tif]

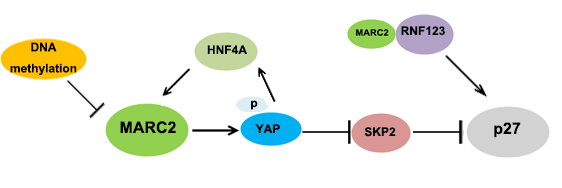

Supplement: Supplementary file 6 — Supplemental Figure 5 [file 41388_2020_1417_MOESM6_ESM.tif]
